# Hunting techniques and their harvest as indicators of mammal diversity and threat in Northern Angola

**DOI:** 10.1007/s10344-021-01541-y

**Published:** 2021-11-06

**Authors:** Nele Teutloff, Paulina Meller, Manfred Finckh, Almeida Segredo Cabalo, Guedes José Ramiro, Christoph Neinhuis, Thea Lautenschläger

**Affiliations:** 1 Department of Biology, Institute of Botany, Faculty of Science, Technische Universität Dresden, 01062 Dresden, Germany; 2 Biodiversity, Evolution & Ecology of Plants, Institute of Plant Science and Microbiology, University of Hamburg, Ohnhorststr. 18, 22609 Hamburg, Germany; 3 University of Kimpa Vita, Rua Henrique Freitas No. 1, Bairro Popular, Uíge, Province of Uíge Angola

**Keywords:** Snares and traps, Bushmeat, Angolan legislation, Socio-ecological conflict, Zoonoses, Biodiversity crisis

## Abstract

Over-exploitation of wildlife especially bushmeat trade is the second most important threat to animal biodiversity. This also applies to Northern Angola but data on bushmeat and hunting techniques for this region are rare. Therefore, we study the most common hunting techniques, frequently captured species, and their economic value, and discuss the local resource use in relation to Angolan law and urgent global crises like the loss of biodiversity, the food supply in South African countries, and the risk of zoonoses. We recorded bushmeat hunting in 27 localities in the province of Uíge, accompanied hunters along their snare lines and interviewed additional 20 locals. Seven main types of snares and traps and their characteristics were defined. Hunters own on average 92 ± 128.7 snares and traps and capture about 25.3 ± 23.6 animals monthly. In total, respondents recognized 28 species of mammals of which one is considered as extinct and two as very rare. The majority of recorded species are hunted regularly. Rodents are most commonly caught followed by primates and duikers. Harvesting rates decrease with species’ body size, leading to high economic value of and achievable prices for rare, large animals. Overall, our results document the hunting pressure on mammals and the persisting popularity of bushmeat in Northern Angola which poses an imminent threat to remaining mammal populations. Moreover, it endangers ecosystem integrity, rural livelihoods, and human health through the risk of new zoonoses. Our findings underscore the urgent need for sustainable solutions. The Angolan government should play a more active role in enforcing existing hunting legislation to reduce illegal bushmeat trade.

**Supplementary information:**

The online version contains supplementary material available at 10.1007/s10344-021-01541-y.

## Introduction

Bushmeat is defined as meat from wild animals and its consumption has probably accompanied human evolution for over 6 million years (Bahuchet 1993; Stanford and Bunn 2001). The negative impact on hunted animals has been long observed and hunting was a main cause of the megafauna extinction in the late Pleistocene (Barnosky et al. 2004). However, over the last century, the global human population has more than tripled. The African population quintupled since 1950 and will probably reach 2.5 billion people in 2050 (Berlin-Institut für Bevölkerung und Entwicklung 2019), causing an increasing demand for meat. As a result, hunting and defaunation currently threaten biodiversity again, as in the Pleistocene, and constitute the main reason for the “empty forest” phenomenon described by Redford (1992) (Wilkie and Carpenter 1999; Robinson and Bennett 2000). IPBES (2019) reported that over-exploitation of wildlife is the second most important driver of biodiversity loss globally. These concerns are particularly relevant for a tropical country like Angola in Southwest-Central Africa, where decades of post-independence civil war have considerably decimated wildlife (Huntley 2017; Daskin and Pringle 2018). Although most Angolans do not live in forests, the consumption of bushmeat is widespread and it also supplies city dwellers. Timber companies facilitate the trade of bushmeat through the development of road infrastructure, and enlarge the hunting area by beating aisles far into the forest which enable access to preserved areas (Laurance et al. 2006).

The close contact to wild animals increases the risk of zoonoses, as the current outbreak of SARS-CoV-2 proves (Akhtar et al. 2020; Jacob et al. 2020; Tiwari et al. 2020). The largest outbreak of Marburg Fever ever reported globally caused 227 victims in the Angolan province of Uíge in 2004/05 (Ndayimirije and Kindhauser 2005). An ongoing outbreak of Ebola fever in neighboring DRC has caused more than 2200 victims so far. For lack of alternatives, many people in Angola rely on bushmeat for consumption or as an economic income and thereby endanger themselves and the ecosystems they live in.

Both, the increasing loss of biodiversity and the global SARS-CoV-2 pandemic calls for a more complete picture of hunting and its consequences. For this reason, research in less described areas is essential. During previous studies in the municipality of Uíge in Northern Angola, the sale of bushmeat on markets and streets was often observed, suggesting that hunting is a widespread activity. Furthermore, snares and traps, which are generally used by hunters, were found occasionally during fieldtrips. However, sustainable hunting is difficult to maintain. The province of Uíge’s population density of 24.3 persons/km^2^ (Censo 2014) significantly exceeds the recommended sustainability threshold of 1 person/km^2^ (Robinson and Bennett 2000). Furthermore, frequently hunted and endangered species like the White-bellied Pangolin exist in the area, under threat by current practices (Kingdon 2015). Lastly, we assume a relationship between a higher hunting success during specific seasons and habitat types, in which snares and traps are deployed.

In order to address these hypotheses, we conducted interviews in rural areas of Uíge and gathered information about hunting techniques and captured mammal species to assess (a) common hunting techniques, (b) hunted species, and (c) their economic value. Our general aim is to describe dimensions of local resource use which lie at the root of urgent global crises against the background of the Angolan hunting legislation.

## Materials and methods

### Study area

**Fig. 1:**
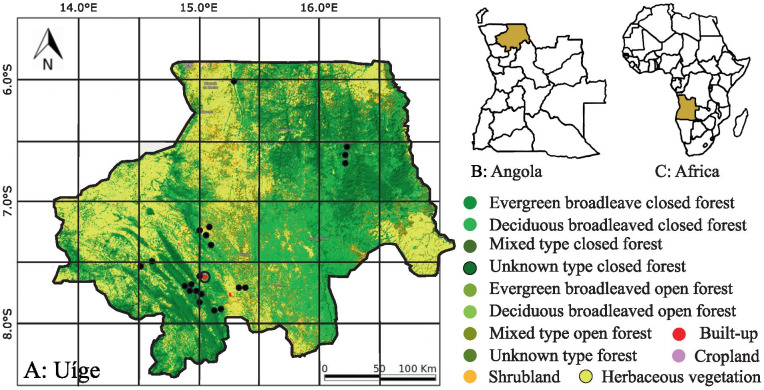
**A** Map of the study area province of Uíge with vegetation zones according to Barbosa (1970), locations of interviews marked in black dots, and Uíge city encircled in black. **B** Province of Uíge located in Angola, **C** Location of Angola in Africa. Graphic: © Copernicus Service Information 2019 (Buchhorn et al. 2020)

From February to April and from October to November 2019, we conducted fieldtrips to 6 municipalities and 27 localities in Angola’s Northern province of Uíge, where surveys were carried out. Research focused mainly on the prospective protected areas: Serra do Pingano, Serra Uíge, Serra Canacanjungo, and Mucaba. Additional studies took place in other municipalities throughout the province of Uíge. The province of Uíge borders in the north and east with the Democratic Republic of the Congo, in the south with the provinces of Malanje, Cuanza Norte, and Bengo, and in the west with the province of Zaire (Fig. 1). It is divided in 16 municipalities with a total area of 58,698 km^2^ (Censo 2014).

The geomorphology of Uíge is described in detail by Huntley et al. (2019): Uíge is divided into the Escarpment Zone to the west, and the Congo Peneplain to the east. The Escarpment Zone is a hilly transition zone between the coastal zone and interior plateaus and has characteristic mountain chains (Serras). These mountain chains dominate the south west of Uíge city, the area where most data were collected, with altitudes up to 1200 m and annual precipitation between 1300 and 1600 mm (Lautenschläger and Neinhuis 2014, 2020). The sandy Congo Peneplain is shaped by many northwards flowing rivers, tributaries of the Congo Basin.

The climate is a tropical wet or dry savannah climate (Aw) based on the Köppen climate classification, and includes a rainy season in the hot wet summer from October to April, with a relative air humidity of 85–90% (Peel et al. 2007; Lautenschläger and Neinhuis 2014; Huntley et al. 2019). The winter is cool and dry with temperatures around 20 °C, lasting from June until September (Huntley et al. 2019).

Angola contains a larger range of biomes, seven out of nine, than any other African country (White 1983; Huntley et al. 2019) and encompasses 15 ecoregions (Olson et al. 2001). The province of Uíge is described by White (1983) as a transition zone between the drier peripheral semi-evergreen Guineo-Congolian rain forest, the Zambezian dry evergreen forest, and transition woodland. According to the more recent classification of ecoregions by Burgess et al. (2004), also used by the World Wildlife Fund (WWF) (Dinerstein et al. 2017), the province of Uíge belongs to the ecoregion ‘Western Congolian Forest-Savannah Mosaic’, a mixture of tropical and subtropical grasslands, savannahs, shrublands and woodlands (Olson et al. 2001; Burgess et al. 2004).

### Methods

Due to the large size and to difficulties in accessibility of the area under research, the methods mainly concentrated on interviews with local hunters and public market surveys. During fieldwork, Portuguese language was mainly used. However, in some cases, Angolan colleagues translated to the local language Kikongo. Gender and age of every respondent was documented wherever possible and localities of interviews were georeferenced. For all fieldtrips, the University Kimpa Vita formulated in advance credentials to inform the mayors of the municipalities about planned research activities. To establish contact with potential respondents, local authorities of the visited villages (soba and seculo) were informed about the aims and methods of the study and asked to suggest persons with experience in hunting that might participate. Methods were based on the techniques developed by Noss (1998a) to answer our central question on:

- *Hunting techniques*: We observed snare hunters and accompanied 23 hunters along their snare lines. We recorded different types of snares and traps, and hunters were asked about their function, characteristics, number of snares, control frequency, capture rate, and the species of captured animals. Further documented information was the time of construction: the actual time needed in the field to either build a snare/trap if self-made or activate it if bought; durability: the time the snare/trap is active in the field before it has to be rebuild; and how the knowledge of constructing snares was passed on. Additionally, hunters stated the location of their snares (savannah, forest or in both habitats equally) and the most profitable season (dry or rainy season, or whether hunting success is independent of the season). Detected snares and traps were classified according to types found in literature: Spring-Loaded Bar Mousetrap, Simple Cable Snares, two types of Foot-Snares, Spring-Spear-Traps, Deadfall Trap and Steel-Leghold Trap (Bateman 1989; Noss 1998b; Proulx 1999; Burr 2015). Additional information was gathered for fishing rods and bird nets. However, we noted variation in snare types and hunting behaviors across distant regions, as a result of adaption to different environmental conditions, the influence of personal skill, and local traditions. Thus, some snares and traps may vary from original descriptions recorded in the literature and were assigned to the most similar type.
- *Species specific information*: We performed interviews with 15 of the 23 accompanied hunters and additional 20 locals by showing pictures of animal species which possibly occur in the region (Kingdon 2015). The respondents were asked about abundance, harvesting rate, local name and economic value of the species. Not all of the 20 locals were still hunting actively, they also included village elders. The economic value of the species was documented in local currency (AOA) between October and November 2019. Because of the strongly fluctuating exchange rate, we applied a standardized average of $ 1 = 430 AOA for our analysis. The number of respondents per interview varies as many were interviewed in groups.
  Regulations for hunting and Angolan law: Hunting is only allowed during the hunting season (1^st^ August – 31^st^ December) with a hunting licence and only for the following reasons: local subsistence (nutrition, clothing, medicine and culture), necessity (control of population growth, protection of goods or self-defence), sports, and scientific research. An additional licence by the IDF (Instituto de Desenvolvimento Florestal) is needed for selling products (Ministério de Ultramar 1955; Ministério da Agricultura do Desenvolvimento Rural e das Pescas 1957; Ministério do Ensino Superior 2017). The Combined Executive Decree No. 37/99 contains a present list of animals whose hunting is prohibited (Annex I) or whose hunting is allowed in specific seasons (Annex II) (Ministry of Agriculture and Rural Development 1999; Jones 2008; Mauck 2013). The additional `Yellow List of Angolan Species` (LEA) categorizes species according to their status of abundance or threat of extinction (Ministério do Ambiente 2018a). Furthermore, Angola is part of CITES (Convention on International Trade in Endangered Species of Wild Fauna and Flora). Appendix I of CITES lists species that are threatened by extinction. CITES prohibits international trade of these species, except when the purpose of the import is not commercial (CITES 2019).
- *Economic value of captured species*: Complementary to field trips, public market surveys were conducted at the principal market in Uíge city, so as to record traded animals and their market value. In addition, sellers were shown on printed images other species, and were asked which ones they usually sell and at which price. This information was complemented by own observations during and between field trips. Identification of recorded animals and data analysis were completed in Dresden, Germany.
  Collected information is based on respondent’s statements, which must be evaluated with care. Recording of private hunting is not common because people mostly hunt illegally and without licences. As a result, many of the hunters interviewed responded reluctantly. Furthermore, secondary education in rural regions is rare, resulting in difficulties for most respondents in making careful assessments regarding time and numbers, such as frequency of harvest. Finally, it is important to note that hunting behavior is performed at irregular time intervals, as many hunters also work in agriculture. Thus, their statements can only approximate reality.

## Results

**Fig. 2:**
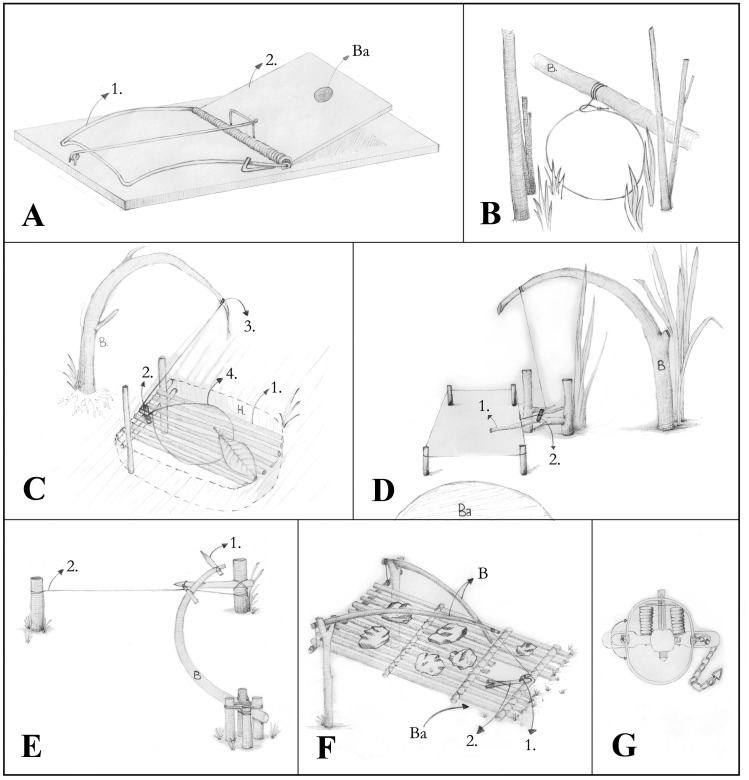
Drawings of observed snare and trap types with their main characteristics. **A** Spring-Loaded Bar Mousetrap, **B** Simple Cable Snare, **C** Foot-Snare Model 1, **D** Foot-Snare Model 2, **E** Spring-Spear Trap, **F** Deadfall Trap, **G** Steel-Leghold Trap. Functions are described in Table 1

**Fig. 3:**
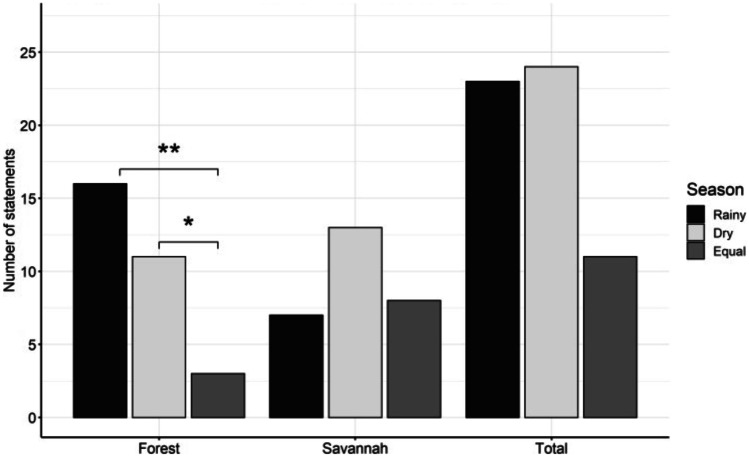
Number of statements related to the location and more profitable season according to each snare and trap types. A significant difference with *p* < 0.05 to *p* = 0.01 is marked with one star and with *p* < 0.01 to *p* = 0.001 is marked with two stars

**Fig. 4:**
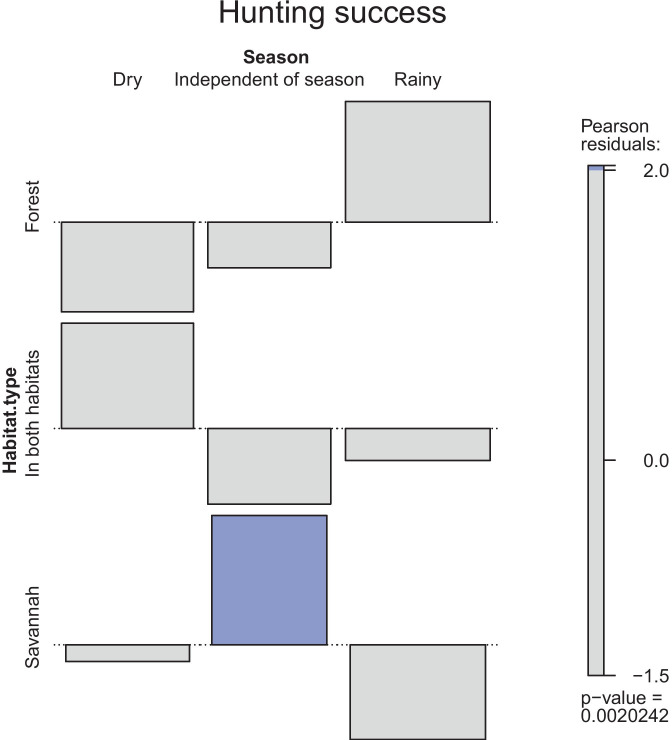
Association plot showing the differences between observed and expected values regarding the chi-square test for the hunting success depending on (1) location of snares: snares located in savannah, forest or located equally in both habitats, and (2) the season: higher hunting success in dry or rainy season or season independent hunting success. Significant relationship with a confidence interval of 0.05 is marked in dark grey. The area of the box is proportional to the difference in observed and expected values. When the box rises above the dotted baseline, the observed value of a cell is greater than the expected one

A) Hunting techniques
  We detected seven main types of snares and traps (Tables 1 and 2, Fig. 2, Online Resource Fig. S1) of which most are nonselective. Some types, however, such as the spring-loaded bar mousetrap target specific species. Specific baits as manioc or mice attract different feeding guilds like herbivores or carnivores. Although fishing rods or barriers, and bird nets are not specifically made to hunt mammals, bats enter nets and otter-shrews rods, accidentally. As they are not set up daily and do not have a permanent intake, they are mentioned in Table 1 but excluded from the average harvesting rate.
  The simple cable snare is the most used snare with on average 80.0 ± 13.6 activated exemplars in the field at a time per hunter. The trap type with the highest capture rate was the spring-loaded bar mousetrap with a mean success rate of 19.0 ± 10.6 animals per month. The knowledge necessary for constructing self-made snares and traps is mostly passed on from previous generations. Only 3 hunters (13%) learnt through observations from friends or unknown people. Regarding the construction/ deployment time, it is important to differentiate between self-made snares and traps, which have to be built in the field, and purchased traps, which only have to be deployed. The most complex self-made trap was the deadfall trap with on average 9.4 h ± 7.2 h construction time, whereas the easiest type was the simple cable snare with on average 7.3 ± 3.1 min construction time. The spring-loaded bar mousetrap and the steel-leghold trap are bought at markets. To deploy them, it requires 1 min or 5.7 ± 4 min, respectively. A spring-loaded bar mousetrap costs on average US$ 0.35 ± 0.12 and a steel-leghold-trap US$ 14.00 ± 6.50. More sophisticated and commercial types of snares and traps (deadfall trap, spring-loaded bar mousetrap, steel-leghold trap) last up to several years, while smaller self-made snares and traps (cable snares, foot snares) are far less durable. For example, the simple cable snare stays only active in the field for 6.3 ± 0.4 months on average. The partly high standard deviations are due to large differences in what hunters reported.
  The distribution of snares and traps used in forest and savannah is virtually the same (forest 52%, savannah 48%; Fig. 3) but the hunting success differed slightly across the habitat types and locations. According to the hunters’ statements, only a slight trend was evident for a greater hunting success depending on habitat type or season (*X*^2^ = 5.899, df = 2, *p* = 0.052). This is reflected in higher hunting success in forests during the rainy season, and in savannahs during the dry season. Hunting success in forests was more influenced by season (*X*^2^ = 8.200, df = 2, *p* = 0.0136) than in savannahs, where the success was rather equal (*X*^2^ = 2.214, df = 2, *p* = 0.331). This finding is supported by the association plot (Fig. 4), showing only one significant association for a season-independent hunting success in savannahs.
  Other habitat types used for hunting are caves where five hunters (10.6%) confirmed using nets to capture bats, and riversides where fishing rods or barriers and some cable snares are located.
  On average, every hunter uses 2.2 ± 1.2 different types and owns 92 ± 128.7 snares and traps (Table 3), whereby the latter varies strongly among the 23 hunters. Consequently, the monthly capture rate varies likewise with an average of 25.3 and a standard deviation of 23.6 animals per month. The interviewed hunters were exclusively male and, according to their statements, they control their snare lines every 2.3 ± 1.1 days. However, in single cases, differences between statements and reality have been observed as visited snares contained trapped animals which were already totally rotten.

**Fig. 5:**
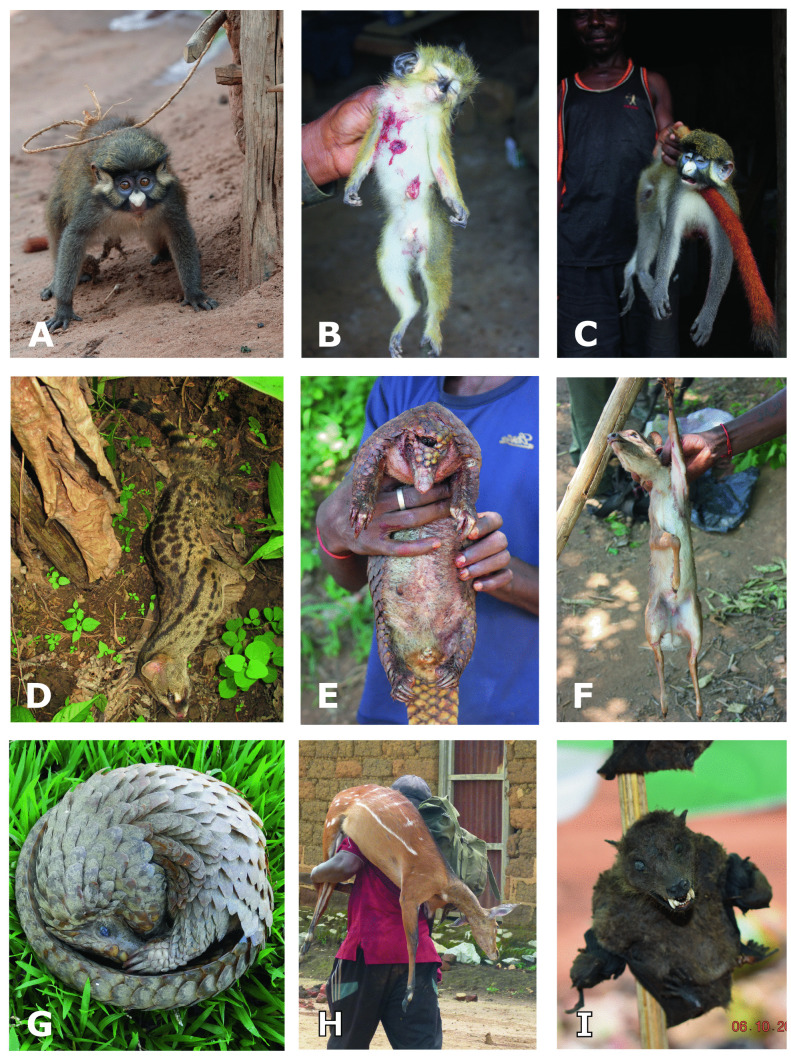
Living, dead, and smoked captured animals; **A**, **C** Red-tailed Monkey (*Cercopithecus a. ssp.*), **B** Southern Talapoin (*Miopithecus talapoin*)*,* **D** Genet (*Genetta sp.*), **E**, **G** White-bellied Pangolin (*Phataginus tricuspis*), **F** Blue Duiker (*Philantomba monticola*) **H** Bushbuck (*Tragelaphus scriptus*), **I** smoked unidentified bat species. Photographs by the authors except: **A** Lucas Lange, **D** Viola Clausnitzer, **G** Anne Göhre

**Table 1:** Commonly used snare and trap types classified in types found in the literature (Bateman 1989; Noss 1998b; Proulx 1999; Burr 2015): average number of snares per hunter for every snare type, average control frequency of snare lines, average of the monthly harvest rate, average distance to the belonging village (in minutes by foot), average time required for construction/deployment, and average durability of snare and trap types. All values with their respective standard deviation. Further information shows the profitable seasons (R rainy season, D dry season, I independent), preferred location (F forest, S savannah, C field, R river, Ca caves), and regions of detected occurrence (N Negage, M Mucaba, MZ Maquela do Zombo, SP Serra do Pingano, K Kimbele, A Ambuila)

**Snares types**	**Average number of snares per hunter**	**Control frequency [every x days]**	**Average capture rate (monthly)**	**Profitable season**	**Region**	**Location**	**Distance to village [in min by foot]**	**Time for construction/deployment**	**Durability**
Spring-Loaded Bar Mousetrap	27.5 ± 7.7	Daily ± 0	19.0 ± 10.6	D, I	N, M	F, S, C		1.0 ± 0.0 min	> 1 year
Simple Cable Snares	79.9 ± 13.6	2.0 ± 1.1	12.4 ± 8.7	R, D, I	N, SP, MZ, K, A	F, S, R	27.0 ± 13.5	7.3 ± 3.1 min	6.3 ± 0.4 months
Foot-Snare: Model 1	29.0 ± 14.4	2.5 ± 1.3	4.0 ± 3.0	R, D	N, SP, MZ, A	F, S	43.1 ± 33.6	51.6 ± 59.0 min	11.3 ± 11.0 months
Foot-Snare: Model 2	40.5 ± 10.2	2.4 ± 1.3	8.5 ± 6.0	R, D, I	N, SP, M, K, MZ	F, S	27.0 ± 19	8.3 ± 2.9 min	4.4 ± 5.8 months
Spring-Spear-Traps	30 ± 0	3.5 ± 1.0		R, D	N, Sp	F, S		15.0 ± 0.0 min	
Deadfall Trap	11.5 ± 12.8	1.6 ± 0.8	10.4 ± 6.1	R, I, D	SP, M, K, A	F, S	23.4 ± 10.4	564 ± 432 min	16.0 ± 6.9 months
Steel-Leghold Trap	2.6 ± 0.6	2.5 ± 1.5	5.0 ± 4.2	R, D, I	SP, N, M	F, C, S	10.0 ± 0	5.7 ± 4.0 min	> 15 years
Fishing Rod or Barrier	1 ± 0	2.3 ± 0.6	98xday	R	SP, N, MZ, K	R	30 ± 0	2.0 ± 0.0 h	
Bird Nets			25xnight	R	SP	F, B, Ca			
On average	35.5 ± 21.4	2.3 ± 1.1	9.6 ± 7.3				26.7 ± 20.4

**Table 2:** Description of Fig. 2 illustrated snare and trap types with their mechanisms, material, and captured species: 1: *Graphiurus* sp*.,* 2: *Lophuromys* sp*.*, 3: *Hylomyscus* sp*.*, 4: *Grammomys* sp*.*, 5: *Funisciurus pyrropus*, 6: *Thryonomys swinderianus*, 7: *Protoxerus stangeri*, 8: *Atherurus africanus*, 9: *Anomalurus* sp*.*, 10: *Cercopithecus ascanius* ssp*.*, 11: *Miopithecus talapoin*, 12: *Colobus angolensis*, 13: *Philantomba monticola*, 14: *Sylvicapra grimmia*, 15: *Cephalophus silvicultor*, 16: *Phataginus tricuspis*, 17: *Potamochoerus* sp*.*, 18: *Genetta* sp*.*, 19: *Mungos Mungo*, 20: *Bdeogale nigripes*, 21: *Crossarchus ansorgei*, 22: *Tragelaphus scriptus*, 23: *Potamogale velox*, 24: *Syncerus caffer nano*, 25: *Loxodonta cyclotis*, 26: *Hypsignathus monstrosus*, 27: *Rousettus aegypticus*, 28: *Myonycteris angolensis*, 29: *Micropteropus pusillus*

**A: Spring-Loaded Bar Mousetrap:**
The mechanism works with a spring-loaded bar (1) and a trip (2) to release it. The spring-loaded bar swings around rapidly with great impact when an animal, usually a mouse, touches the trip. By the force of the bar the mouses’ neck breaks. The trip can contain a bait (Ba) like manioc
Material: wood, metal, bait
Captured animal species: 1, 2,3,4, 7, 9
**Cable Snares**
**B: Simple Cable Snares:** An open cable noose, vertical and above the ground is fixed to a branch (B) or similar. By entering, the cable noose tightens and closes around the neck of the animal. The cable noose can be set up in different sizes depending on the hunted animal species
Material: cord (bought or self-made of natural fibers), nylon, branches
Captured animal species: 1, 2, 3, 4, 5, 6, 7, 8, 9, 10, 11, 13, 14, 15, 16, 17, 18, 19, 20, 21, 22, 23, 24, 28, 29
**C: Foot-Snare Model 1:**
A small hole (H) is covered with a platform of bark, small sticks and leaves which acts as a trigger mechanism. A cable noose encircles the edge of the hole and is attached to a bent-over branch (B). When an animal steps on the platform (1) stick 1 gets released (2). The branch springs up (3) and tighten the cable noose around the leg of the animal (4)
Material: cord, branches
Captured animal species: 1, 2, 3, 4, 5, 6, 7, 8, 9, 10, 11, 13, 14, 15, 16, 17, 18, 19, 20, 21, 22, 23, 24
**D: Foot-Snares Model 2:**
Water in which fishes were gutted serves as a bait (Ba) by filling it into a hole in front of the snare. When the animal steps in the snare on stick 1, the trigger mechanism and stick 2 get released. The bent-over branch (B) pulls backward and tighten the cable noose around the animals’ limb. There is a variation of this model designed for birds where the trigger mechanism gets activated when a bird touches stick 1 with the intention of finding a place to sit
Material: baits (manioc, mice, fishwater), cord, branches
Captured animal species: 1, 2, 3, 4, 5, 6, 7, 8, 9, 10, 11, 13, 14, 16, 17, 18, 19, 20, 21, 22
**E: Spring-Spear-Traps:**
A spear (1) attached to a bent branch (B) impales whatever is in its path when the trigger in form of a stretched cord (2) is activated
Material: cord, branches
Captured animal species: 1, 2, 3, 4, 5, 6, 7, 9, 13, 14, 16, 18, 19, 20, 21, 22
**F: Deadfall Trap:**
A platform of branches carrying stones hangs on two bent-over branches (B) which are attached to a cord. The cord connects the branches with a little stick 1 clamped between the platform and a stick 2, which touches the ground. A bait (Ba) under the platform like manioc or fishwater attracts animals and by searching for food they move stick 2 what releases stick 1. The two branches spring backward and the platform falls down onto the animal
Material: Trunks, branches, cord, stones, bait
Captured animal species: 1, 2, 3, 4, 5, 6, 7, 9, 10, 11, 13, 16, 18, 19, 20, 21
**G: Steel-Leghold Trap**:
Steel-Traps are made of metal and were first imported to Africa by colonists from Europe to hunt large predators while exploiting tropical countries in the late eighteenth century (Bateman 1989). The trap consists of two jaws opened to 180° at set position and each closes 90° upon each other when an animal steps on the trap
Material: Steel, sometimes with additional branches
Captured animal species: 1, 2, 3, 4, 5, 6, 7, 8, 9, 10, 11, 13, 14, 16, 17, 18, 19, 20, 21, 22

**Table 3:** Average number of snare or trap types per hunter, average number snares and traps per hunter, and average capture rate per month per hunter. Additionally, the average age of hunters and the gender of all hunters. All values with their respective standard deviation

Average number of snare or trap types per hunter	2.2 ± 1.2
Average number of snares and traps per hunter	92.1 ± 128.7
Average capture rate per hunter (animals per monthly)	25.3 ± 23.6
Average age of hunters in years	38.5 ± 13.3
Proportion of male hunters in %	100

**Table 4:** According to respondent’s statements, the recognized animal species were divided into existing, extinct or rarely seen, regularly hunted, and rarely hunted species in the four prospective protected areas

Number of:	**Serra do Pingano**	**Serra Uíge**	**Serra Canacanjungo**	**Mucaba**
Stated existing species	23	14	23	24
Stated extinct or rarely seen species	4	10	3	3
Stated regularly hunted species	22	2	7	23
Stated rarely or casually hunted species	2	8	10	1

**Table 5:** Documented mammal species according to group and ordered by harvesting rate. Status of species regarding to hunting legislation season after Angolan Law (Decree No. 37/99) (App. I – Forbidden to hunt, App. II – Allowed in announced season), the threat assessed by Angolan government in LEA (categorized in A – Extinct, B – Threatened with Extinction, C – Vulnerable, D – Abundant, E – Important Species (because of culture, endemic, tourism etc.), IUCN and CITES (LC – Least Concern, D – Decreasing, NT – Near Threatened, V – Vulnerable, E – Endangered). The average harvesting rate is given in animals captured per hunter per year or per net and night – p.n., Average sales prices of the species in US$ provided by the hunters

**Group**	**Nb**	**Scientific name**	**English name**	**Decree No. 37/99/ LEA**	**IUCN/ CITES status**	**Harvesting rate per year/ per capture event**	**Value in US$**
Rodents	1	*Graphiurus sp.*	African Dormice		LC	3723 ± 3444 p.y	0.25 ± 0.09
	2	*Lophuromys sp.*	Brush-furred Mice		LC	2112 ± 3639 p.y	0.22 ± 0.05
	3	*Hylomyscus sp.*	African Wood Mice		LC	2605 ± 2782 p.y	0.20 ± 0.06
	4	*Grammomys sp.*	Narrow-footed Thicket Rats		LC	2605 ± 2782 p.y	0.20 ± 0.06
	5	*Funisciurus pyrropus*	Fire-footed Rope Squirrel		LC	221.3 ± 309.0 p.y	0.91 ± 0.88
	6	*Thryonomys swinderianus*	Marsh Cane Rat		LC	138.3 ± 104.9 p.y	14.41 ± 8.27
	7	*Protoxerus stangeri*	African Giant Squirrel		LC	80.2 ± 79.2 p.y	0.55 ± 0.42
	8	*Atherurus africanus*	Brush-tailed Porcupine	App. II	LC	92.6 ± 95.2 p.y	5.81 ± 1.64
	9	*Anomalurus sp.*	Anomalure		LC	60.3 ± 48.6 p.y	5.48 ± 4.18
Primates	10	*Cercopithecus ascanius ssp.*	Red-tailed Monkey	C	LC, D	71.0 ± 106.3 p.y	9.48 ± 2.51
	11	*Miopithecus talapoin*	Southern Talapoin	D	V	46.8 ± 15.2 p.y	8.50 ± 1.80
	12	*Colobus angolensis*	Angola Colobus	App. II/ B	LC	36.0 ± 12.0 p.y	10.47 ± 5.32
Duiker	13	*Philantomba monticola*	Blue Duiker	App. II/ D	LC, D/ App. II	50.9 ± 53.3 p.y	8.94 ± 2.44
	14	*Sylvicapra grimmia*	Bush Duiker	App. II/ B	LC, D	49.0 ± 51.7 p.y	62.58 ± 33.56
	15	*Cephalophus silvicultor*	Yellow-backed Duiker	App. I	NT/ Ap. II	2.0 ± 0.0 p.y	151.16
Pangolin	16	*Phataginus tricuspis*	White-bellied Pangolin	App. I	E/ App. I	60.4 ± 58.7 p.y	5.81 ± 1.21
Pigs	17	*Potamochoerus sp.*	Bushpig	App. II/ D	LC	22.3 ± 15.3 p.y	58.14 ± 21.5
Carnivores	18	*Genetta sp.*	Genet	App. II	LC	46.8 ± 38.7 p.y	4.65 ± 2.23
	19	*Mungos Mungo*	Banded Mongoose	App. II	LC	44.0 ± 36.8 p.y	7.98 ± 3.86
Horned-Antelopes	22	*Tragelaphus scriptus*	Bushbuck	App. II/ D	LC	30.0 ± 0.0 p.y	35.70 ± 22.69
Otter-shrew	23	*Potamogale velox*	Giant Otter-shrew	App. II	LC, D	21.5 ± 20.5 p.y	3.26 ± 1.70
Oxen	24	*Syncerus caffer nano*	African Forest Buffalo	App. II/ B, E	NT	–	–
Elephant	25	*Loxodonta cyclotis*	Forest Elephant	App. I/ C	V/ App. I	–	–
Bats	26	*Hypsignathus monstrosus*	Hammer-Headed Fruit Bat		LC	21.8 ± 18.3 p.n	2.47 ± 3.08
	27	*Rousettus aegypticus*	Egyptian Rousette		LC	20.2 ± 17.7 p.n	0.23 ± 0.00
	28	*Myonycteris angolensis*	Collared Fruit Bats		LC	13.5 ± 13.3 p.n	0.34 ± 0.14
	29	*Micropteropus pusillus*	Dwarf Epauletted Fruit Bats		LC	–	–
	30	*Rhinolophus sp*	Horseshoe Bats		LC	–	–

B) Species specific information and C) their economic value
  A total of 28 species were identified by the respondents, of which 27 species were reported to still occur in the study areas. The Forest Buffalo (*Syncerus caffer nanus*) was described as extinct; all statements agree on its previous presence, but it hasn’t been seen for years. The Forest Elephant (*Loxodonta cyclotis*) and the Angola Colobus (*Colobus angolensis*) were mostly described as nearly extinct or very rarely seen. The Forest Elephant was not seen for an undefined period of time but respondents stated its appearance in 2018 between Serra do Pingano and Serra Uíge, and 2019 in a village adjacent to Serra Canacanjungo. The occurrence of the rare primate Angolan Colobus was only reported from respondents of Mucaba.
  The statements regarding the occurrence of the Ansorge’s Cusimanse (*Crossarchus ansorgei*) and the Mongoose (*Bdeogale nigripes*) varied significantly and made a valid statement difficult. Therefore, they are excluded from Tables 4 and 5.
  Many respondents did not differentiate between different bat species (Chiroptera). Furthermore, bats were captured with nets, which are not set up daily. Both factors made it difficult to identify the hunted species and provide the respective capture rates. However, some species with characteristic traits could be identified and some species were observed on markets and at villages for sale. Only these species are included in Tables 4 and 5. Table 4 summarizes the statements of the respondents regarding their observation of species occurrence. A selection of captured species is shown in Fig. 5.
  While most species are captured regularly, some animals are trapped occasionally or accidentally like the Giant Otter-shrew (*Potamogale velox*), which entangles in fishnets. In the Serra Uíge, it was stated that only two species are hunted regularly and eight occasionally. However, we only found one hunter who was willing to answer our survey leading to little information and most probably, more than two species are hunted as snares and traps are nonselective.
  According to the assessment by the Angolan government, three species are threatened by extinction: the Angola Colobus (*Colobus angolensis*), the Bush Duiker (*Sylvicapra grimmia*), and the African Forest Buffalo (*Syncerus caffer nanus*) (Table 5). Furthermore, it is prohibited by law through the Decree No. 37/99 to hunt the following three species: the Yellow-backed Duiker (*Cephalophus silvicultor*), the White-bellied Pangolin (*Phataginus tricuspis*), and the Forest Elephant (*Loxodonta cyclotis).* According to CITES, the Yellow-backed Duiker and the African Forest Buffalo are near-threatened, the Forest Elephant and the Southern Talapoin are vulnerable, and the White-bellied Pangolin is endangered (CITES 2019).
  The highest harvesting rates were documented for small rodents and bats with several individuals per day or night. The lowest harvesting rates belong to the Yellow-backed Duiker, the Bushbuck and the Giant Otter-shrew. The hunters did not report any captures of the African Forest Buffalo, the Forest Elephant, or the Horseshoe Bat. The hunters’ sales prices for the captured species are summarized in Table 5. The values vary widely between US$ 0.20 ± 0.06 for a small rodent and US$ 58.14 ± 21.50 for a bushpig. Data obtained from the market analysis is relatively limited and can be summarized as follows: Most common on markets are species with a medium body size and a relatively high value like duiker and primates (the Blue Duiker and Red-tailed Monkey were observed at all market visits) followed by larger rodents like the Marsh Cane Rat (were observed at half of the visits). The White-bellied Pangolin, the Bushpig, and different bat species like the Hammer-Headed Fruit Bat were only observed few times. All other species were not seen during the market analysis.

## Discussion

A) Hunting techniques
  **Relationship between costs, material availability, capture rates, and the frequency of usage of snares and traps**
  The two factors influencing the hunters’ use of snare and trap types, and their respective numbers are mainly complexity (effort and time of construction) and cost (purchasability at markets). Complex snares and traps need more construction time and the number owned per hunters is relatively low compared to quickly constructed snares. Industrially produced traps can be expensive, e.g., the steel-leghold trap can cost up to US$ 23.3 which is a high economic investment. For the self-made simple cable snare, only a cord is needed, which is often made by the hunters out of natural fibers with high tensile strength like *Triumfetta cordifolia* or *Urena lobata* (Senwitz et al. 2016). Both factors could explain the particularly high number of cable snares of on average 79.9 ± 13.6 per hunter compared to only around 2.6 ± 0.6 steel-leghold traps per hunter. Some snares and traps are non-selective, allowing to capture larger animal species with higher economic value, which could be another factor influencing the choice of hunters.
  **Mismatch between statements and reality on snare line revisions and its implications on animal welfare**
  According to hunter’s statements, snare lines are controlled regularly. However, these statements might be overconfident as we visited snares in which trapped animals were already totally rotten. Our observation is confirmed by previous research. Noss (1998b) reports that 25% of the total number of captures were decomposed or eaten by scavengers in the hunters’ absence. Furthermore, many animals can escape from snares with serious injuries and often die as a result. A survey in the Central African Republic showed that one third of all animals caught by cable snares broke the cable and escaped with an injury (Noss 1998b). Thus, the number of animals actually dying or suffering because of hunting is much higher than the reported capture rates. As a result, 129 countries worldwide have banned or restricted the use of snare and traps (The Law Library of Congress 2016). Especially, the use of leghold traps is prohibited in many countries because captured animals either die slowly and painful from exposure, shock, thirst or loss of blood, or survive with serious injuries (Michaud 1997).
  **Relationship between hunting success and snare location and season**
  The hunting success is equally high in savannahs throughout the year, and slightly season-dependent in forests (Figures 3 and 4). Likely, different species are targeted in each habitat and season; otherwise, we would expect a much stronger seasonal/spatial pattern. Consequently, certain species are always hunted in each season and habitat, resulting in high hunting success and hunting pressure in all habitats and seasons, giving the animals little chance for recovery. Based on these findings, a detailed species-based investigation on hunting success is urgently needed. The results could be helpful to identify seasons and habitats of high hunting pressure for endangered species in order to protect them through targeted regulations.
  **Further hunting methods**
  During the interviews, many hunters explained further hunting methods like fumigating caves or blocking the entrance with stones. As mentioned before, the focus was on hunters using traditional methods due to vague and unreliable statements from hunters with shotguns. However, Bersacola (2014) found that 84% of fresh carcasses sold on roads in central Angola are hunted with shotguns and only 16% are trapped using metal or string snares. Larger species such as primates are preferably hunted with shotguns, while snares mainly capture smaller animals like blue duiker (Bersacola et al. 2014). Thus, the use of shotguns causes a much higher hunting pressure for large (and rare) species than snares. In Monte Alén National Park in Equatorial Guinea, hunting with shotguns almost caused the local extinction of the black colobus monkeys (*Colobus satanas*) (Kümpel et al. 2008). The private ownership of guns for hunting purpose is only allowed with a licence and it is forbidden to hunt at night, the preferred time for hunting with shotguns, which makes access to information difficult (Ministério da Agricultura do Desenvolvimento Rural e das Pescas 1957). However, this information would be highly valuable as shotguns probably contribute to a significant amount to the existing hunting pressure on mammals in Angola, and would therefore result in different target species and capture rates.
B) Species specific information and Angolan law
  Not all species that are caught are also sold as bushmeat on markets or along roads. Mostly rodents, followed by primates and duikers, are hunted, while market interviews imply the opposite tendency: mostly duiker, primates, and tall rodents were observed on markets. In numbers, more than 96 % of captured animals are rodents, but few were documented as bushmeat on markets. Similarly, Bersacola et al. (2014) show that the public bushmeat trade in Central Angola consists primarily of duikers (53.6 %) and primates (12.7 %). It seems like smaller animals are used for direct and local consumption while for larger animals, it is worth transporting them to markets in cities. However, it is important to keep in mind that on markets, the harvest of all hunting methods is mixed, including shotgun hunting, which probably further increases the differences in range and quantity between market sales and hunters’ harvest.
  In total, hunting is prohibited by law for only three of the documented species, although five species are assessed as threatened by extinction or vulnerable by the Angolan government (Ministry of Agriculture and Rural Development 1999; Ministério do Ambiente 2018a). Hunting of the near-threatened (IUCN) Yellow-backed Duiker is officially forbidden, but in fact poorly controlled (Ministry of Agriculture and Rural Development 1999; Bersacola et al. 2014). For example, it is still permitted to hunt the African Forest Buffalo at announced seasons, although it is categorized as “Threatened with Extinction.” It nowadays exists only in two small areas, while being extinct in the rest of Angola (Ministry of Agriculture and Rural Development 1999; Kingdon 2015). This seems paradoxical as it is planned to establish a protected area especially for the preservation of the African Forest Buffalo (Presidente da Republica Angolana 2020). Furthermore, we observed a mismatch between Angola’s’ governmental assessment of the Bush Duiker and its appearance on markets and hunting rates. It seems to be relatively abundant as it is a commonly hunted and sold duiker while the government assesses it as threatened with extinction. However, this assessment is explained by the intense poaching of this species focusing on the threat for the Bush Duiker rather than on actually low population rates (Ministério do Ambiente 2018b).
  Out of the four African pangolin species, the endangered (IUCN) White-bellied Pangolin(Phataginus tricuspis) is still the most common, but also the most hunted. In Ghana, the White-bellied Pangolin represented 82 % of the observed pangolins traded by the stakeholders (Boakye et al. 2016). It is currently estimated that 0.4–2.7 million pangolins are hunted annually only in Central African forests, representing an increase of around 150 % over the past four decades (Ingram et al. 2018). The White-bellied Pangolin is extensively captured not only for bushmeat but also for their scales for traditional medicine, and the illegal trade to Asian markets (listed in App. I of CITES). Another threat for the pangolin is the habitat loss through agriculture, which is reflected in declining populations in many parts of its occurrence(Jansen et al. 2020).
C) Economic value of captured species
  Traditionally, bushmeat is an important source of animal protein for the majority of rural families in the nearby Congo Basin (Wilkie and Carpenter 1999; Nasi et al. 2011). In Gabonese households, it can make up 10 % of all meat-based meals, whereas in Malawi up to 39 % of the population regularly consumes bushmeat (Bachand et al. 2015; van Velden et al. 2020). The price of bushmeat depends mainly on the species. Species like smaller rodents and bats with high reproduction rates are caught most frequently with several individuals per day or night. With growing body size, the harvesting rates decline to few individuals per month or year. This is reflected in prices: frequently hunted and smaller species are cheaper (US$ 0.19 ± 0.06–0.28 ± 0.09 per mouse) than rarer species (up to US$ 151.16 per Yellow-backed Duiker). Furthermore, it depends on the species if bushmeat has a similar or more expensive price than meat from domestic animals. For instance, the Blue Duiker costs in average US$ 8.94 ± 2.44 with a body weight between 3.5 and 9 kg (Kingdon 2015). Prices for goats reach up to US$ 40 with an average body weight of 45 kg (unpub., TU Dresden, Mileski 2004). Thus, the Blue Duiker would be slightly more expensive but in a similar price category while the very rarely hunted Yellow-backed Duiker with 45–80 kg costs more than the triple to fivefold (Bersacola et al. 2014; Kingdon 2015). However, bushmeat consumption is not only popular because of affordable prices or non-affordable alternatives (Fa et al. 2002; Wilkie et al. 2005). In fact, urban families that could afford alternative meat often regard bushmeat as a ‘treat’ for special occasions (van Vliet and Mbazza 2011)
  Examples from Equatorial Guinea and Malawi show that consumption is predominantly driven by availability and preference for taste of wild meat and increased diet diversity (Fa et al. 2002; van Velden et al., 2020). However, alternatives are rare in rural areas in Northern Angola, where animals usually are kept privately and domestication at larger scale is limited. Livestock must be fed and recurring diseases reduce numbers drastically (unpub., TU Dresden). Thus, access to livestock meat is not always possible and costly, whereas sourcing bushmeat is relatively easy when owning a gun or knowing how to construct snares.
  Economically, hunting can contribute substantially to daily income or compensate crop failures of families living in rural areas (Wilkie and Carpenter 1999; Nasi et al. 2011; van Velden et al. 2020). The majority of people in the area of the province of Uíge works in subsistence agriculture (80 %) and a normal monthly income ranges between US$ 43.9 for collecting plants, US$ 46.5 for agriculture, and US$ 83.7 for the production of charcoal (Nienguesso 2020). In comparison, one Yellow backed duiker has a value of US$ 151.16. Bersacola et al. (2014) also recorded prices ranging between app. $ 5 for a 0.7-kg African giant squirrel to app. $ 250 for a 65-kg Yellow backed duiker. Bushmeat prices therefore make hunting and trading attractive, which puts additional pressure on already rare and endangered species.
D) Methodological approach and limitations of the survey
  Using interviews as a research method and the selection of the respondents have clearly impacted the results. First, it was decided to focus only on hunters using traditional hunting methods like snares and traps because the ownership and use of shotguns is mostly prohibited in Angola causing limited responses from hunters. However, this selectivity limits the survey as only a part of the hunters and hunting methods are documented and discussed. For example, larger species such as primates are preferably hunted with shotguns, while snares mainly capture smaller animals like small rodents (Bersacola et al. 2014). Consequentially, the species captured and the capture rates stated in this survey are directly influenced by the methodology used. Second, the small sample size (23 hunters and 20 locals) of the survey leaves the results susceptible for biased responses. Among the interviewed hunters, hunter’s behavior varied widely in, e.g., hunting frequency, location of snares, and snare numbers. Some respondents hunt occasionally, while others hunt on a daily basis, leading to a wide range in number of snares per hunter as well as in capture rates. Although the location of the snares not necessarily has an influence in the hunting success, it will affect the kind of species captured. The high standard deviation throughout the data reflects this variety giving single extreme statements a great weight. Third, bushmeat is mainly directly consumed or sold in villages, on markets and in restaurants. This analysis covered a great part of bushmeat consumed in villages and sold on markets while the share of bushmeat sold in restaurants was not explicitly examined. A part of the bushmeat hunted with snares and traps is probably sold to restaurants. However, we are missing details on the ratio between self-consumption and sales in villages, on markets or restaurants.
E) Local resource use and global crises
  Unsustainable hunting pressure at the local level is closely linked to critical global issues:
  1. Biodiversity: obviously, bushmeat consumption can lead to population decline and (local) extinction of hunted species (Redford 1992). Hunting indirectly impacts other species within the same ecosystem, thereby impairing, e.g., seed dispersal (Wright 2003). Primates and large mammals are particularly relevant as dispersers for large seeded plant species (Kingdon 2015). The real impact of hunting in the province of Uíge is difficult to estimate because of unknown local population sizes and rates. Currently, some species like the White-bellied Pangolin suffer unsustainable harvest rates given the already low population densities. The population density of the White-bellied Pangolin ranges between 0.68 and 0.84 individuals/km^2^ (Cameroon and Benin) with a harvest rate of 30 per year by a single hunter in the province of Uíge (Akpona et al. 2008; Bobo et al. 2014). The decline of mammal biodiversity in the province of Uíge is significant and unique species like the White-bellied Pangolin and the endemic Southern Talapoin are likely to disappear in the near future. It is doubtful whether defaunated ecosystems will be able to support ecosystem services and livelihoods as before.
  2. Poverty: commercial hunters in the Central African Republic can earn up to 700 US$ per year. Such wages are significantly above the average income (Noss 1998a) incentivizing for excessive hunting. At the same time, bushmeat constitutes a source of essential proteins and amino acids for remote communities, not easily replaceable by plants and livestock (Bennett 2002; Wilkie et al. 2016). Particularly children of up to four years suffer from malnutrition in Angola, e.g., causing 14.7% of child deaths in the municipality of Dande (Rosário et al. 2016). While overhunting mitigates to a certain degree the adverse effects of rural poverty, it simultaneously destroys its own resource base and, thus, affects the livelihoods of people depending on bushmeat as a source of protein. Commercial hunting, in particular, is not a sustainable solution but a vicious circle with adverse effects for local communities.
  3. Health: The current outbreak of the coronavirus SARS-CoV-2 and the overall increased occurrences of zoonosis in the past 30 years illustrates the high risk emerging from a close contact to wild animals. At the whole-genome level, SARS-CoV-2 is 96% identical to a bat coronavirus detected in *Rhinolophus affinis* (Horseshoe Bat), a species belonging to the family of Rhinolophidae known in Angola, too (Kingdon 2015; Guo et al. 2020; Zhou et al. 2020). These bats are regularly hunted and consumed around the province of Uíge. Frequently hunted (and commercialized) bats from the province of Uíge are known as hosts of critical transmittable diseases like Ebola (Hammer-Headed Fruit Bat) and Marburg virus (Egyptian Rousette). In Uíge, the largest outbreak of Marburg Fever ever reported globally caused 227 victims in 2004/05 (Ndayimirije and Kindhauser 2005) and the outbreak of Ebola in the neighboring DRC in 2018 is still not completely under control. Thus, handling and consumption (particularly of bats and primates) includes a high health risk for transmittable diseases like Ebola, HIV, or coronaviruses (Jones et al. 2008; Guo et al. 2020). The current practice of bushmeat hunting and commodification in Northern Angola is therefore of high risk, in particular for poor rural and urban populations with limited access to medical care.

We describe a complex interdependence of “traditional” natural resource extraction under rapidly changing socio-economic settings and increasing demographic pressure, declining biodiversity, and unresolved health risks in a small part of Africa. The current system is no longer sustainable and solutions are urgently needed to reduce excessive hunting pressure, protect endangered species, constrain the risk of zoonoses, and improve alternative protein supply for rural families. Angolas’ urban population has alternative supply options to replace the favored but not essential bushmeat with other animal protein. An important starting point would be to suppress roadside commodification of bushmeat. Trade and transport of bushmeat to urban areas need strict legal and sanitary controls. Subsistence hunting should be restricted to rural self-supply and education is required related to the consumption of known disease vectors. Establishing protected areas could help populations of overharvested species to recover. However, these approaches are doomed to fail without law enforcement and societal participation (Chêne 2010). The responsible Angolan authorities need to pay greater attention to the illegal trade with endangered species and enforce existing Angolan legislation and international obligations with respect to hunting. Reducing bushmeat trade will bring important benefits for the county: it will reduce health risks for vulnerable groups of the population, it will help to maintain the long-term resource base for rural subsistence hunting, and it will stabilize a wide spectrum of forest dependent ecosystem functions and services. However, ultimately, the Angolan people need to be the drivers of such sustainable stewardship of ecosystems and biodiversity.

## Supplementary Information

Below is the link to the electronic supplementary material.

Supplementary file1 (DOCX 2477 KB)

Supplementary file2 (DOCX 34 KB)

## Data Availability

All data are available from the corresponding author.
