# Supplementary material for: Hunting techniques and their harvest as indicators of mammal diversity and threat in Northern Angola
Source: Eur J Wildl Res. 2021 Nov 6;67(6):101. doi: 10.1007/s10344-021-01541-y (PMC8572081; doi:10.1007/s10344-021-01541-y)
Supplement: Supplementary file 1 — Supplementary file1 (DOCX 2477 KB) [file 10344_2021_1541_MOESM1_ESM.docx]

**Appendix I (Online Resource)**


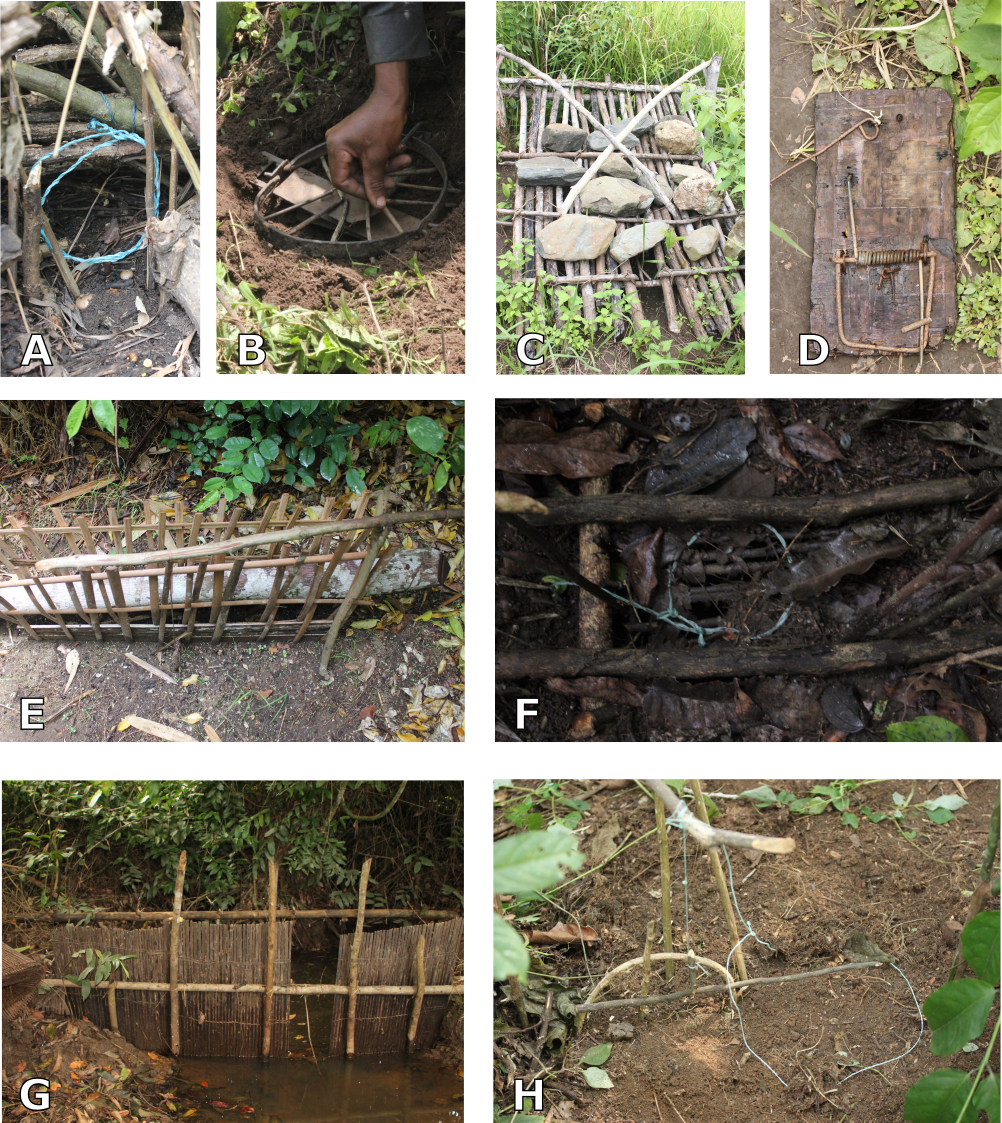
 **Figure S1**: Observed snares and traps: A: Simple Cable Snare, B: Steel-Leghold Trap, C: Deadfall Trap, D: Spring-Loaded Bar Mousetrap, E: Deadfall Trap, F: Foot-snare Model 1, G: Fish Barrier, H: Foot-snare Model 2. Photographs by the authors except: B: Christin Heinze

**Table S1:** Snare types sorted according to literature (Bateman 1989), region of detected occurrence, average number of snares per hunter for every snare type, control frequency of snare lines and average of monthly harvest rate. Further information shows preferred location of snares (F-Forest, S-Savannah, C-Field, R-River, B-under Bridges, Ca-Caves), species captured with snare type (Number according to Appendix II) and profitable seasons (R-Rainy season, D-Dry season, I-Independent).

| **Snares Types** | **Region** | **Average number of snares per hunter** | **Control frequency [every x days]** | **Harvest rate (monthly)** | **Location** | **Captured animal species** | **Profitable season** |
| --- | --- | --- | --- | --- | --- | --- | --- |
| Spring-loaded Bar Mousetrap | Serra Canacanjungo | 22 | daily | 8-12 | F,S | 1, 2, 3, 4, 7, 9 | D |
|  | Mucaba | 33 | daily | 28 | S,C | 1, 2, 3, 4 | I |
| Simple Cable Snares | Serra Canacanjungo | 300 | 3-4 | 24 | F, S | 1, 2, 3, 4, 5, 6, 7, 8, 9, 11, 13, 14, 16, 17, 18, 19, 22, 26, 28, 29 | R, D |
|  | Serra Pingano | 31 | 2-3 | 12 | F, S | 1, 2, 3, 4, 5, 6, 7, 8, 9, 10, 11, 13, 14, 15, 24, 16, 17, 18, 19, 20, 21, 22, 23, 26 | R |
|  | Maquila do Zombo | 40 | daily | 1 | F, S | 6, 22 | D |
|  | Kimbele | 8-9 | 2-3 | 8 | S, R | 1, 2, 3, 4, 5, 6, 7, 9, 10, 11, 13, 16, 17, 18, 19, 20, 21, 22, 23 | D, I |
|  | Ambuila | 20 | daily | 17 | F, S | 6, 8, 19, 20 | I |
| Foot-snare | Serra Canacanjungo | 10 | 3-5 | 2-3 | F | 6, 7, 8, 9, 11, 13, 14, 16, 17, 18, 22 | R |
|  | Serra Pingano | 31 | 3 | 4 | F, S, C | 1, 2, 3, 4, 5, 6, 7, 8, 9, 10, 11, 13, 14, 15, 16, 17, 18, 19, 20, 21, 22, 23, 24 | R |
|  | Maquila do Zombo | 30 | daily | <1 | F, S | 6, 22 | D |
|  | Ambuila | 45 | 2 | 7 | F, S | 1, 2, 3, 4, 8, 17, 22 | D |
| Spring-snares | Serra Pingano | 25 | 4 | 3-4 | F, S | 1, 2, 3, 4, 5, 6, 7, 8, 9, 10, 13, 14, 16, 17, 18, 19, 20, 21 | R |
|  | Mucaba | 28 | 2 | app. 14 | F, S, C | 1, 2, 3, 5, 4, 6, 7, 9, 10, 11, 16, 19, 20, 21 | R, D |
|  | Kimbele | 9 | 2-3 | 10 | F, S | 1, 2, 3, 4, 5, 6, 7, 9, 16, 18, 19, 20, birds | D, R, I |
|  | Maquila do Zombo | 100 | daily | 14 | S | 6 | D |
|  | Negage |  |  | 1 | S | 8, 11, 13, 14, 16, 17, 22 | I |
| Spring-spear-traps | Serra Canacanjungo | 30 | 4 | 1 | F, S | 1, 2, 3, 4, 5, 6, 8, 11, 13, 14, 16, 17, 18, 19, 22 | D, I |
|  | Serra Pingano |  | 2-3 |  | F, S | 2, 3, 4, 5, 6, 7, 9, 16, 18, 19, 20, 21 | R |
| Deadfall Trap | Serra Pingano | 30 | daily | 16 | F | 2, 3, 4, 5, 6, 9, 10, 11, 13, 16, 18, 20, 21 | R |
|  | Mucaba | 3 | daily | 3-4 | S | 1, 2, 3, 4, 6, 21 | I |
|  | Kimbele | 10 | 2-3 | 15 | F, S | 1, 2, 3, 4, 5, 6, 7, 16, 18, 19, 20 | I |
|  | Ambuila | 3 | 2 | 7 | S | 6 | D |
| Steel-Leghold Trap | Serra Pingano | 3 | 2-3 | 2 | F, C | 1, 2, 3, 4, 5, 6, 7, 8, 9, 10, 11, 13 14, 16, 17, 18, 19, 20, 22 | R |
|  | Serra Canacanjungo | 2 | 4 |  | F | 1, 2, 3, 4, 5, 6, 13, 14, 16, 18, 19, 22 | D |
|  | Mucaba | 3 | daily | 8 | S, C | 5, 6, 7, 9, 10, 11, 16, 19, 20, 21 | I |
| Fishing Rod or Barrier | Serra Pingano |  | 2 | 10-15xday |  | unidentified |  |
|  | Serra Canacanjungo |  |  | 50xday | R | unidentified | R |
|  | Maquila do Zombo | 1 |  | 30-200xday | R | unidentified |  |
|  | Kimbele | 1 | 2-3 | 100xday | R | unidentified |  |
| Bird Nets | Serra Pingano |  |  | 25xnight | F, B, Ca | 9, 27, 28, 29 | R |
| Average per snare | In total | 32 - 33 | 2 | 9-10 | S(21), F(20), C(5), R(4), Ca (1) |  | D(12), R(12), I(8) |
