# Supplementary material for: Hunting techniques and their harvest as indicators of mammal diversity and threat in Northern Angola
Source: Eur J Wildl Res. 2021 Nov 6;67(6):101. doi: 10.1007/s10344-021-01541-y (PMC8572081; doi:10.1007/s10344-021-01541-y)
Supplement: Supplementary file 2 — Supplementary file2 (DOCX 34 KB) [file 10344_2021_1541_MOESM2_ESM.docx]

**Appendix II**

**Table S2:** Documented mammal species according to species and group and ordered by harvesting rate. For the areas of Serra do Pingano and Serra Uíge the abundance given in number of statements, the harvest rate given in number of hunted animals and the value in Angolan currency (Thousand AOA [TAOA], in Oct.-Nov. 2019) are presented. Numbers belong to Appendix I.

| Nb | Scientific Name | Serra do Pingano | | | | | | | | | | Serra Uíge | | | | | | | |
| --- | --- | --- | --- | --- | --- | --- | --- | --- | --- | --- | --- | --- | --- | --- | --- | --- | --- | --- | --- |
|  |  | Abundance | | | | | Harvest rate | | | | Sales value from hunter [TAOA] | Abundance | | | | | Harvest rate | | |
|  |  | frequent | medium | rare | doesn´t exist anymore | not known/ never existed | daily hunted | regularly hunted (monthly) | rarely hunting (Av. per year) | casualy hunted (in lifetime) |  | frequent | medium | rare | doesn´t exist anymore | not known | daily hunted | rarely hunting (Av. per year) | casualy hunted (in lifetime) |
| 1 | *Graphiurus* sp. | 7 | 1 |  |  | 1 | 20-30 | 5-6 |  |  | 0,1 |  |  |  |  |  |  |  |  |
| 2 | *Lophuromys* sp. | 8 | 1 | 2 |  |  | 15 | 17-18 |  |  | 0,1 | 1 |  |  |  |  |  |  |  |
| 3 | *Hylomyscus* sp. | 7 | 2 |  |  |  | 10 | 5-6 |  |  | 0,1 |  | 1 |  |  |  |  |  |  |
| 4 | *Grammomys* sp. | 6 | 2 |  |  |  | 10 | 5-6 |  |  | 0,1 |  |  |  |  |  |  |  |  |
| 5 | *Funisciurus pyrropus* | 9 | 2 |  |  |  | 2-3 | 4-5 |  |  | 0,2 | 1 |  |  |  |  |  |  |  |
| 6 | *Thryonomys swinderianus* | 10 |  | 1 |  |  | 3 | 7-8 |  |  | 7 | 1 |  |  |  |  |  | x |  |
| 7 | *Protoxerus stangeri* | 4 | 2 |  |  | 5 |  | 6-7 |  |  |  | 1 |  |  |  |  |  |  |  |
| 8 | *Atherurus africanus* | 4 | 2 |  |  | 4 |  | 4 | 4 |  | 2,5 |  | 1 |  |  |  |  |  |  |
| 9 | *Anomalurus* sp. | 5 | 1 | 1 |  | 2 |  | 8-9 | 1 |  | 2,75 |  |  | 1 |  |  |  |  |  |
| 10 | *Cercopithecus ascanius* ssp. | 8 | 2 |  |  | 1 | 1 | 2-3 |  |  | 4 | 1 |  |  |  |  |  | 1-2 |  |
| 11 | *Miopithecus talapoin* | 7 | 2 |  | 1 |  |  | 2-3 |  |  | 3,5 | 1 |  |  |  |  |  |  |  |
| 12 | *Colobus angolensis* |  |  |  | 3 | 8 |  |  |  |  |  |  |  |  |  | 1 |  |  |  |
| 13 | *Philantomba monticola* | 8 | 2 |  |  |  |  | 7-8 | 3-4 |  | 4 | 1 |  |  |  |  |  | 4-5 |  |
| 14 | *Sylvicapra grimmia* | 6 | 2 | 3 |  |  |  | 7-8 |  |  | 32 |  | 2 |  |  |  |  | 2 |  |
| 15 | *Cephalophus silvicultor* |  |  | 4 | 2 | 5 |  |  |  | 1 | 65 |  |  | 1 |  |  |  | 2 |  |
| 16 | *Phataginus tricuspis* | 6 | 1 | 2 | 2 |  |  | 11 | 5 | 1 | 2,5 | 1 |  |  |  |  |  | 3-4 |  |
| 17 | *Potamochoerus* sp. | 4 | 1 | 2 | 1 |  |  | 3 | 1 | 1 | 28 |  | 2 |  |  |  |  |  |  |
| 18 | *Genetta* sp. | 2 | 4 | 3 |  | 2 |  | 3-4 |  | 1-2 | 2 |  |  | 1 |  |  |  |  |  |
| 19 | *Mungos mungo* | 5 | 1 |  | 1 | 3 |  | 3-4 |  |  | 3,75 |  | 1 |  |  |  |  |  |  |
| 20 | *Bdeogale nigripes* | 3 | 2 | 2 | 1 | 2 |  | 1-2 |  |  | 3,5 | 1 |  |  |  |  |  |  |  |
| 21 | *Crossarchus ansorgei* | 3 | 1 |  |  | 7 |  |  |  | x |  |  |  | 1 |  |  |  |  | x |
| 22 | *Tragelaphus scriptus* | 4 | 4 | 2 |  |  |  | 2-3 |  |  | 40 |  |  | 1 |  |  |  |  | x |
| 23 | *Potamogale* sp*.* | 4 | 3 | 1 |  | 1 |  |  | 1 | 2 | 2 |  |  | 1 |  |  |  |  |  |
| 24 | *Syncerus caffer nanus* |  |  | 2 | 9 |  |  |  |  |  | 200 |  |  |  | 1 |  |  |  |  |
| 25 | *Loxodonta cyclotis* |  |  | 1 | 3 | 6 |  |  |  |  |  |  |  |  |  | 1 |  |  |  |
| 26 | *Hypsignathus monstrosus* | 1 |  | 2 |  | 1 | 40 |  |  |  | 0,12 |  |  | 1 |  |  |  |  |  |
| 27 | *Rousettus aegypticus* | 3 | 2 |  |  |  | 45 |  |  |  | 0,1 |  | 1 |  |  |  |  |  |  |
| 28 | *Myonycteris torquata* | 8 | 1 |  | 1 |  | 20 |  |  |  | 0,1 |  |  | 1 |  |  | 4-5 |  |  |
| 29 | *Micropteropus pusillus* | 8 | 1 |  | 1 |  | 20 |  |  |  | 0,1 |  |  | 1 |  |  | 4-5 |  |  |
| 30 | *Rhinolophus* sp. |  |  |  |  | 5 |  |  |  |  |  |  |  |  |  | 1 |  |  |  |

**Table S3:** Documented mammal species according to species and group and ordered by harvesting rate. For the areas of Serra Canacanjungo and Mucaba the abundance given in number of statements, the harvest rate given in number of hunted animals and the value per animal in Angolan currency (Thousand AOA [TAOA], in Oct.-Nov. 2019) are presented. Numbers belong to Appendix I.

| Nb. | Scientific Name | Serra Canacanjungo | | | | | | | | | | Mucaba | | | | | | | | | | Sales value at principal market, Uíge [mil kwz] |
| --- | --- | --- | --- | --- | --- | --- | --- | --- | --- | --- | --- | --- | --- | --- | --- | --- | --- | --- | --- | --- | --- | --- |
|  |  | Abundance | | | | | Harvest rate | | | | Sales value from hunter [TAOA] | Abundance | | | | | Harvest rate | | | | Sales value from hunter [TAOA] |  |
|  |  | frequent | medium | rare | doesn´t exist anymore | not known | daily hunted | regularly hunted (monthly) | rarely hunting (Av. per year) | casualy hunted (in lifetime) |  | frequent | medium | rare | doesn´t exist anymore | not known | daily hunted | regularly hunted (monthly) | rarely hunting (Av. per year) | casualy hunted (in lifetime) |  |  |
| 1 | *Graphiurus* sp. | 2 |  |  |  |  |  | x |  |  | 0,18 | 2 |  |  |  |  | 8-9 |  |  |  | 0,08 |  |
| 2 | *Lophuromys* sp. | 2 |  |  |  |  | 1 |  |  |  | 0,1 | 2 |  |  |  |  | 3-4 |  |  |  | 0,08 |  |
| 3 | *Hylomyscus* sp. |  | 1 |  |  |  |  |  |  |  |  | 1 |  |  |  |  | 4-5 |  |  |  | 0,05 |  |
| 4 | *Grammomys* sp. |  | 1 |  |  |  |  |  |  |  |  | 1 |  |  |  |  | 4-5 |  |  |  | 0,05 |  |
| 5 | *Funisciurus pyrropus* | 1 |  |  |  |  |  |  |  |  |  | 2 |  |  |  |  |  | 20 |  |  | 0,5 |  |
| 6 | *Thryonomys swinderianus* | 2 |  |  |  |  |  | ~1 |  |  | 9 | 2 |  |  |  |  |  | 16 |  |  | 3 | 3-8 |
| 7 | *Protoxerus stangeri* | 1 |  |  |  |  |  |  | 5 |  | 0,18 | 2 |  |  |  |  |  | 18 |  |  | 0,13 |  |
| 8 | *Atherurus africanus* |  | 2 |  |  |  |  |  | 3 |  | 3,5 | 2 |  |  |  |  |  | 16 |  |  | 2,5 | 1,5-3 |
| 9 | *Anomalurus* sp*.* | 2 |  |  |  |  |  |  |  | x | 1,75 | 1 | 1 |  |  |  |  |  | x |  | 5 |  |
| 10 | *Cercopithecus ascanius* | 2 | 1 |  |  |  |  | 4 |  |  | 3,5 | 1 |  | 1 |  |  |  | 10 | 1 |  | 3 | 3,5-6 |
| 11 | *Miopithecus talapoin* | 2 |  |  |  |  |  | 4 |  |  | 3,5 | 2 |  |  |  |  |  | 4 |  | 1 | 3 |  |
| 12 | *Colobus angolensis* |  |  |  |  | 2 |  |  |  |  |  | 2 |  |  |  |  |  | 3 |  |  | 3 |  |
| 13 | *Philantomba monticola* | 1 | 1 |  |  |  |  | 1 |  |  | 4 | 1 | 1 |  |  |  |  | 3-4 |  |  | 2,5 | 4-6 |
| 14 | *Sylvicapra grimmia* |  | 2 |  |  |  |  | 1 |  |  | 19 |  | 2 |  |  |  |  | 1 |  |  | 25 | 15-25 |
| 15 | *Cephalophus silvicultor* |  |  | 1 |  |  |  |  |  |  |  |  |  |  | 1 | 1 |  |  |  |  |  |  |
| 16 | *Phataginus tricuspis* |  | 1 | 1 |  |  |  |  | 2-3 |  | 3 | 1 | 1 |  |  |  |  | 4-5 | 1 |  | 2,5 | 1,5-3 |
| 17 | *Potamochoerus* sp. | 1 | 1 |  |  |  |  |  |  | x | 27 | 1 | 1 |  |  |  |  | 2-3 |  |  | 35 | 10-30 |
| 18 | *Genetta* sp. | 1 | 1 |  |  |  |  |  |  | x | 2 | 1 |  | 1 |  |  |  | 6 | 1 |  | 2,3 | 2 |
| 19 | *Mungos Mungo* |  | 1 |  |  |  |  |  |  |  |  | 1 |  | 1 |  |  |  | 1-2 |  | 1 | 3 |  |
| 20 | *Bdeogale nigripes* |  | 1 |  |  |  |  |  |  |  |  |  |  | 1 |  |  |  | 4 |  |  | 3 | 2-3 |
| 21 | *Crossarchus ansorgei* |  |  |  |  | 1 |  |  |  |  |  |  |  |  |  | 2 |  |  |  |  |  |  |
| 22 | *Tragelaphus scriptus* |  | 2 |  |  |  |  | 1 |  |  | 20 |  | 2 |  |  |  |  | 1 |  | 1 | 25 | 6-18 |
| 23 | *Potamogale* sp. | 2 |  |  |  |  |  |  |  | x | 0,75 | 2 |  |  |  |  |  | 3-4 |  | 1 | 1,5 |  |
| 24 | *Syncerus caffer nanus* |  |  | 1 | 1 |  |  |  |  |  |  |  |  |  | 2 |  |  |  |  |  |  |  |
| 25 | *Loxodonta cyclotis* |  |  | 2 |  |  |  |  |  |  |  |  |  |  | 1 | 1 |  |  |  |  |  |  |
| 26 | *Hypsignathus monstrosus* |  | 1 |  |  |  |  |  |  |  |  |  | 2 |  |  |  | 3-4 |  |  |  |  | 0,2 |
| 27 | *Rousettus aegypticus* |  | 1 |  |  |  |  |  | 3-4 |  |  | 2 |  |  |  |  | 7 |  |  |  | 0,23 | 0,2 |
| 28 | *Myonycteris torquata* | 2 |  |  |  |  |  |  | 4-5 |  | 0,1 | 2 |  |  |  |  | 7 |  |  |  | 0,23 | 0,2 |
| 29 | *Micropteropus pusillus* | 2 |  |  |  |  |  |  | 4-5 |  | 0,1 | 2 |  |  |  |  | 7 |  |  |  | 0,23 | 0,2 |
| 30 | *Rhinolophus* sp. |  |  |  |  | 1 |  |  |  |  |  |  |  |  |  | 1 |  |  |  |  |  |  |

**Table S4:** Documented mammal species ordered by group with English name and mostly used names in the research area in Portuguese and native languages. For one species can exist various local names because different areas were observed and names can double as differentiation was partly hard for locals.

| **Group** | **Scientific Name** | **English Name** | **Most common used local Names** | |
| --- | --- | --- | --- | --- |
|  |  |  | **in portuguese** | **in native languages** |
| Rodents | *Graphiurus* sp. | African Dormice | Rato | Kinze, Kumbi, Nkuzu, |
|  | *Lophuromys* sp. | Brush-furred Mice | Rato | Mbubu, Ngoni, Kinze, MBubuzala |
|  | *Hylomyscus* sp*.* | African Wood Mice | Rato | Kinze/a, Lukinzu, Mbala Mbala |
|  | *Grammomys* sp. | Narrow-footed Thicket Rats | Rato | Mbende, Ngondi, Kinza, Mbala Mbala |
|  | *Funisciurus pyrropus* | Fire-footed Rope Squirrel | Esquilo vermelho | Kikuto/ Ikutu, Kasa, Lubengo, Kaluhaki |
|  | *Thryonomys swinderianus* | Marsh Cane Rat |  | Cambuige, Xixi |
|  | *Protoxerus stangeri* | African Giant Squirrel | (Rato) Esquilo | Ohadi, (Di)kasa, |
|  | *Atherurus africanus* | Brush-tailed Porcupine | Porco espinho | Kizaka, Nsiekele, Ourixo |
|  | *Anomalurus* sp. | Anomalure |  | Pengi/a, Duhadi |
| Primates | *Cercopithecus ascanius* ssp. | Red-tailed Monkey | Macaco vermelho | Kima, Kehu |
|  | *Miopithecus talapoin* | Southern Talapoin | Macaco verde, Macaco casthano | Kima, Kehu, Kunduko, Mudondo |
|  | *Colobus angolensis* | Angola Colobus | Macaco branco | Kipengi, Kimbungo |
| Duiker | *Philantomba monticola* | Blue Duiker | Gazela | Cecha/ Cexi |
|  | *Sylvicapra grimmia* | Bush Duiker | Cabra do mato | Kimpiti |
|  | *Cephalophus silvicultor* | Yellow-backed Duiker | Burro do Mato | Kahi, Vanda, Mbungo, Cuba |
| Pangolin | *Phataginus tricuspis* | White-bellied Pangolin | Pangolin | Kaka, Pregisa/ Pregisoso |
| Pigs | *Potamochoerus* sp. | (Bushpig) | Javali, Porco do Mato | Ngulu Mfuti, Ngulu Zeka |
| Carnivores | *Genetta* sp. | Genet | (Bataca) | Nbongi, Mbala, Kalombongue |
|  | *Mungos Mungo* | Banded Mongoose | Mungos mungo, Canta pedra,  cão da mata | Ntoto, Nfuki, Nsiekele, Mfuengue |
|  | *Bdeogale nigripes* | Mongoose | Rato monteiro | Fuenga, Mkenga |
| Horned-Antelopes | *Tragelaphus scriptus* | Bushbuck | Veado | Mubuengo-Gulungo, Nsá |
| Otter-shrew | *Potamogale* sp. | Giant Otter-shrew |  | Luzoizoi, Lunzundu, Ngola, Kaluçuia |
| Oxen | *Syncerus caffer nano* | African Forest Buffalo | Búfalo | Pacassa |
| Elephant | *Loxodonta cyclotis* | Forest Elephant | Elefante | Nsamba |
| Bats | *Hypsignathus monstrosus* | Hammer-Headed Fruit Bat | morcego | Ngembo, Madima, Boi, Ngonzo |
|  | *Rousettus aegypticus* | Egyptian Rousette | morcego | Ngembo |
|  | *Myonycteris torquata* | Collared Fruit Bats | morcego | Ngembo |
|  | *Micropteropus pusillus* | Dwarf Epauletted Fruit Bats | morcego | Ngembo |
|  | *Rhinolophus* sp | Horseshoe Bats | morcego |  |
